# Association of the lupus low disease activity state (LLDAS) with health-related quality of life in a multinational prospective study

**DOI:** 10.1186/s13075-017-1256-6

**Published:** 2017-03-20

**Authors:** Vera Golder, Rangi Kandane-Rathnayake, Alberta Yik-Bun Hoi, Molla Huq, Worawit Louthrenoo, Yuan An, Zhan Guo Li, Shue Fen Luo, Sargunan Sockalingam, Chak Sing Lau, Mo Yin Mok, Aisha Lateef, Kate Franklyn, Susan Morton, Sandra Teresa V. Navarra, Leonid Zamora, Yeong-Jian Wu, Laniyati Hamijoyo, Madelynn Chan, Sean O’Neill, Fiona Goldblatt, Mandana Nikpour, Eric Francis Morand, for the Asia-Pacific Lupus Collaboration

**Affiliations:** 1 Monash University School of Clinical Sciences at Monash Health, Level 5, Block E, Monash Medical Centre, 246 Clayton Road, Clayton, VIC 3168 Melbourne, Australia; 2 The University of Melbourne, Melbourne, Australia; 3 Chiang Mai University Hospital, Chiang Mai, Thailand; 4 People’s Hospital Peking University Health Sciences Center, Beijing, China; 5 Chang Gung Memorial Hospital, Guishan Township, Taiwan; 6 University of Malaya, Kuala Lumpur, Malaysia; 7 University of Hong Kong, Pokfulam, Hong Kong; 8 National University Hospital, Singapore, Republic of Singapore; 9 Monash Health, Melbourne, Australia; 10 University of Santo Tomas Hospital, Manila, Philippines; 11 University of Padjadjaran, Bandung, Indonesia; 12 Tan Tock Seng Hospital, Singapore, Republic of Singapore; 13 University of New South Wales, Sydney, Australia; 14 Royal Adelaide Hospital, Adelaide, Australia

**Keywords:** Systemic lupus erythematosus, Health-related quality of life, Patient-reported outcomes, Treatment target, Low disease activity

## Abstract

**Background:**

Systemic lupus erythematosus (SLE) is associated with significant impairment of health-related quality of life (HR-QoL). Recently, meeting a definition of a lupus low disease activity state (LLDAS), analogous to low disease activity in rheumatoid arthritis, was preliminarily validated as associated with protection from damage accrual. The LLDAS definition has not been previously evaluated for association with patient-reported outcomes. The objective of this study was to determine whether LLDAS is associated with better HR-QoL, and examine predictors of HR-QoL, in a large multiethnic, multinational cohort of patients with SLE.

**Methods:**

HR-QoL was measured using the Medical Outcomes Study 36-item short form health survey (SF-36v2) in a prospective study of 1422 patients. Disease status was measured using the SLE disease activity index (SLEDAI-2 K), physician global assessment (PGA) and LLDAS.

**Results:**

Significant differences in SF-36 domain scores were found between patients stratified by ethnic group, education level and damage score, and with the presence of active musculoskeletal or cutaneous manifestations. In multiple linear regression analysis, Asian ethnicity (*p* < 0.001), a higher level of education (*p* < 0.001), younger age (*p* < 0.001) and shorter disease duration (*p* < 0.01) remained significantly associated with better physical component scores (PCS). Musculoskeletal disease activity (*p* < 0.001) was negatively associated with PCS, and cutaneous activity (*p* = 0.04) was negatively associated with mental component scores (MCS). Patients in LLDAS had better PCS (*p* < 0.001) and MCS (*p* < 0.001) scores and significantly better scores in multiple individual SF-36 domain scores. Disease damage was associated with worse PCS (*p* < 0.001), but not MCS scores.

**Conclusions:**

Ethnicity, education, disease damage and specific organ involvement impacts HR-QoL in SLE. Attainment of LLDAS is associated with better HR-QoL.

**Electronic supplementary material:**

The online version of this article (doi:10.1186/s13075-017-1256-6) contains supplementary material, which is available to authorized users.

## Background

Systemic lupus erythematosus (SLE) is a chronic multisystem autoimmune disease resulting in significant morbidity and reduced quality of life. With the improvement in overall survival of patients with SLE compared to historical outcomes [1], a growing number of young adults face the burden of chronic disease, which includes not only the activity of the disease itself, the adverse effects of treatment and the complications such as organ damage [2], but also the impact of disease on physical function, quality of life and employment. Health-related quality of life (HR-QoL) is a multi-dimensional construct that evaluates different health perceptions and self-reported functional status, and is often included as a key patient-reported outcome (PRO) in studies of chronic disease.

Both generic and disease-specific instruments have been developed to facilitate measurement of PROs, resulting in an increase in the number of studies assessing HR-QoL in SLE [3–6]. PROs are increasingly recognized as an integral part of assessment in clinical trials and in routine practice [7, 8], as they measure domains not captured by physician-assigned disease activity scores. Patients with SLE perform poorly on HR-QoL measures when compared to the general population [9], especially those with concomitant fibromyalgia [10] or fatigue [6, 11]. The effects of SLE on HR-QoL are comparable to other chronic diseases such as chronic heart failure, coronary artery disease, end-stage airways disease, human immunodeficiency virus and rheumatoid arthritis [12–14]. In addition, it has been reported that patients with SLE feel misunderstood by their families, the community and even the specialists treating them [15]. Consequently, patients feel that their quality of life needs are not being met by treating teams [16, 17].

As recently highlighted, measures of a treatment outcome status for use in clinical trials, or in treat-to-target strategy studies, have been lacking in SLE [18, 19]. Definitions of remission may be too stringent for use in routine practice or clinical trials [20], highlighting the need for a definition of low disease activity [18, 19]. Recently, we reported the definition and preliminary validation of a lupus low disease activity state (LLDAS), combining disease activity and treatment domains, attainment of which was shown in a longitudinal cohort study to be protective against damage accrual [21]. For such a measure to have value in clinical practice and clinical trials, it should be associated not only with physician-applied measures of disease activity and damage, but also with PROs. The objectives of this study were to determine whether LLDAS is associated with better HR-QoL, and to determine other predictors of HR-QoL in a large multiethnic multinational cohort of patients with SLE.

## Methods

### Study population

Ten centers from seven countries took part in this study. Patients over the age of 18 years, who fulfilled the classification criteria for SLE (either the 1997 American College of Rheumatology (ACR) criteria [22] or the 2012 Systemic Lupus International Collaborating Clinics (SLICC) criteria [23]) were eligible. The study centers are members of the Asia Pacific Lupus Collaboration (APLC), involved in a multicenter prospective longitudinal study of SLE outcomes; data reported here represent all patients with complete data acquisition from the enrollment visit. Data collection took place between May 2013 and August 2015, during the routine ambulatory care of each patient, using either a standardized paper or electronic case report form.

### Measurement of HR-QoL

HR-QoL was measured using the Medical Outcomes Study 36-item short form health survey (SF-36v2) [24], a generic instrument validated in a number of SLE observational cohorts and clinical trials, and validated in each of the languages used by patients in this study [3, 4, 10, 13, 25, 26]. The SF-36 comprises eight domains including physical function (PF), role physical (RP), bodily pain (BP), general health (GH), vitality (VT), social function (SF), role emotional (RE) and mental health (MH), and two summary scores defined as the physical component score (PCS) and mental component score (MCS). The individual domain scores are expressed on a scale of 0 to 100, and the component summary scores are standardized around a USA normal population mean of 50, with higher scores representing better HR-QoL.

### Other variables

Demographic information, disease characteristics and data on clinical variables were collected from each patient at the study visit date. Demographic variables included gender, ethnicity (self-reported based on the Australian Standard Classification of Cultural and Ethnic Groups [27]), date of birth, year of SLE diagnosis, smoking status, and highest-attained education level. Disease manifestations were determined from the ACR and SLICC classification criteria [22, 23], recorded at study entry on an ever-present basis. Current doses of glucocorticoids and immunosuppressive medications were recorded for each patient. Disease activity was measured using the SLE disease activity index (SLEDAI-2 K) [28], with specific organ system activity derived from components of the SLEDAI-2 K.

Additional disease status measures included a physician global assessment (PGA) of disease activity on a scale of 0 to 3 [29], and fulfillment of the criteria for LLDAS [21]. The operational definition of LLDAS is fulfilled when all of the following criteria are met: (1) SLEDAI-2 K ≤4, with no activity in major organ systems (renal, central nervous system (CNS), cardiopulmonary, vasculitis or fever) and no hemolytic anemia or gastrointestinal activity; (2) no new features of lupus disease activity compared to the previous assessment; (3) a Safety of Estrogens in Lupus Erythematosus National Assessment (SELENA)-SLEDAI PGA (scale 0–3) ≤1; (4) a current prednisolone (or equivalent) dose ≤7.5 mg daily and (5) well-tolerated standard maintenance doses of immunosuppressive drugs and approved biologic agents, excluding investigational drugs. Disease flares compared to the previous visit were measured using the SELENA-SLE flare index (SFI) [29]. Irreversible disease damage was measured using the SLICC damage index (SLICC-DI) [30].

### Data analysis

Pooled cross-sectional data from all centers were analyzed using STATA v13 (StataCorp, College Station, TX, USA). Individual domain and component summary scores are expressed as median and interquartile range, as the data were not normally distributed. To allow for linear regression analysis, domain and summary scores were log-transformed prior to inclusion into models in order to fulfill the assumption of a normal distribution. The exponentiated regression coefficients (coeff) are reported in results for ease of clinical interpretation. This represents (coeff-1)*100% increase or decrease in PCS or MCS scores for every one-unit change in continuous independent variables or a change in category for categorical independent variables.

Variables with a *p* value ≤0.1 in simple linear regression analysis were checked for multicollinearity prior to inclusion into backward stepwise multiple linear regression models for PCS and MCS scores. LLDAS is a composite measure comprising the SLEDAI, PGA, flare index, prednisolone dose and medication use. In addition to assessing the relationship between LLDAS and HR-QoL (model 1), a separate multiple linear regression model was used to ascertain to what degree individual LLDAS components contributed to this relationship (model 2). A third model of the LLDAS components was also tested, but using organ system activity rather than the total SLEDAI-2 K score (model 3). Model adequacy was evaluated using adjusted *R* ^2^, residual and normality plots.

## Results

### Demographic and disease characteristics

A total of 1422 patients were studied. The majority of patients were female (93%), with a mean (±SD) age at diagnosis of 31.2 (±12.2) years and mean (±SD) disease duration of 9.2 (±7.7) years. Caucasians formed 8% of the sample, with the rest of the patients representing Asian ethnicities native to the region (Table 1). Other demographic characteristics are also shown in Table 1. More than half of patients had a history of malar rash, arthritis, hematologic or immunologic manifestations, and 46% had a history of renal disease (Additional file 1: Table S1). The median score in the SLEDAI-2 K was 4 (IQR 2–6). There were 369 patients (26%) with active renal disease, 273 (19%) with cutaneous activity and 119 (8.4%) with musculoskeletal activity; 593 patients (42%) fulfilled criteria for LLDAS (Table 1). The median SLICC-DI score was 0 (IQR 0–1), with 498 patients (35%) having some damage (SLICC-DI >0).

**Table 1:** Patient demographics and disease characteristics

	Number (%) or mean (SD) or median (IQR: 25th–75th)
Country, *n* (%)	
Australia	217 (15%)
China	222 (16%)
Indonesia	98 (7%)
Philippines	124 (9%)
Singapore	219 (15%)
Taiwan	294 (21%)
Thailand	250 (18%)
Ethnicity, *n* (%)	
Caucasian	116 (8%)
Chinese	699 (49%)
Filipino	132 (9%)
Indonesian	101 (7%)
Thai	254 (18%)
Malay	37 (3%)
Vietnamese/Cambodian	22 (2%)
Indian/Sri Lankan	35 (2%)
Other^a^	28 (2%)
Gender, *n* (%)	
Female	1329 (93%)
Highest attained education level^b^	
Primary	241 (17%)
Secondary	548 (38%)
Tertiary	607 (42%)
Age at diagnosis (years)	31.1 (12.2)
Disease duration (years)	9.2 (7.7)
SLICC-DI score	0 (0–1)
Damage present^c^	498 (35%)
PGA at enrollment	0.5 (0.2–1)
Mild flare	170 (12%)
Severe flare	100 (7%)
SLEDAI-2 K	4 (2–6)
Current CNS activity^d^	9 (0.6%)
Current vasculitis^d^	23 (1.6%)
Current renal activity^d^	369 (25.9%)
Current musculoskeletal activity^d^	119 (8.4%)
Current cutaneous activity^d^	273 (19.2%)
Current serositis^d^	12 (0.8%)
Lupus low disease activity state (LLDAS)	593 (42%)
Number (%) of patients taking prednisolone^g^	1167 (82%)
Taking immunosuppressant^e^	762 (53.5%)
Taking antimalarial^f^	1044 (73.3%)

^a^Other includes Hispanic, African, other South-East Asian, Pacific Islander and mixed ethnicity. ^b^Percent present shown in table, percent absent and missing not shown in table. ^c^SLICC-DI >0. ^d^Active based on non-zero SLEDAI-2 K scores in organ domains as indicated. ^e^Either methotrexate, azathioprine, mycophenolate, leflunomide, cyclosporine, cyclophosphamide (in the last 6 months), rituximab (in the last 6 months) and/or belimumab (in the last 6 months). ^f^Either hydroxychloroquine or chloroquine. Abbreviations: *SLE* systemic lupus erythematosus, *SLEDAI* SLE disease activity index, *SLICC* Systemic Lupus International Collaborating Clinics, *DI* damage index, *PGA* physician global assessment, *CNS* central nervous system, *CVA* cerebrovascular accident

^g^Mean dose (SD) 12 mg (13.7)

Individual domain and component summary scores of the SF-36v2 are presented in Table 2. Overall, domains with the highest (best) median, IQR (25th–75th) scores included physical functioning (85, 65–95), role physical (75, 50–100), role emotional (83.3, 58.3–100), and social functioning (75, 50–100). The lowest (worst) medians were observed in vitality (62.5, 50–75) and general health (57, 40–72).

**Table 2:** Short form-36 domain and component summary scores

	Median (IQR: 25th–75th)
Physical functioning	85 (65–95)
Role physical	75 (50–100)
Bodily pain	74 (51–84)
General health	57 (40–72)
Vitality	62.5 (50–75)
Social functioning	75 (50–100)
Role emotional	83.3 (58.3–100)
Mental health	70 (56–80)
Physical component summary score	49.73 (42.74–54.67)
Mental component summary score	48.34 (40.7–53.32)

### Determinants of HR-QoL

**Fig. 1:**
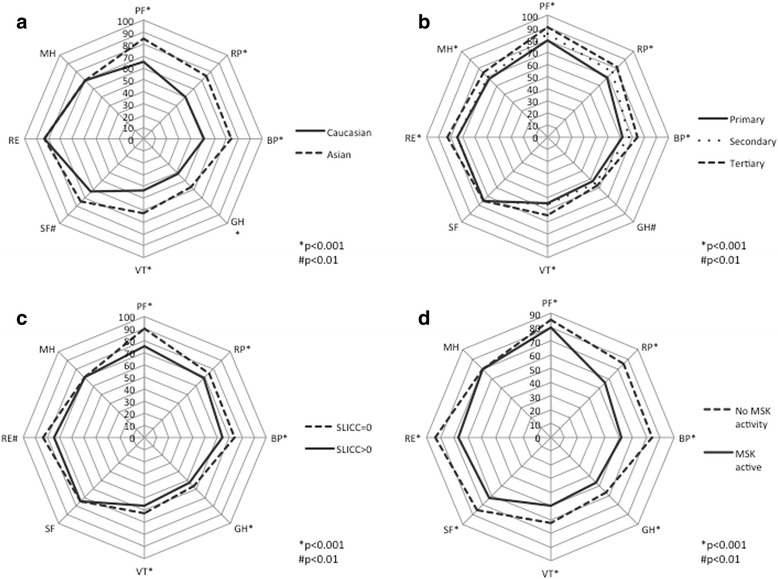
Radar charts comparing short form-36 (SF-36) domain median scores between Asian and Caucasian ethnicity (**a**), primary, secondary and tertiary education levels (**b**), presence (Systemic Lupus International Collaborating Clinics damage index (*SLICC*)-damage index (DI) >0) and absence (SLICC-DI = 0) of disease damage (**c**) and presence and absence of musculoskeletal (*MSK*) activity - either arthritis or myositis on the systemic lupus erythematosus disease activity index-2 K (SLEDAI-2 K) (**d**). Each *spoke* on the radar chart represents an SF-36 domain on a scale of 0–100, with higher scores representing better health-related quality of life. The domains are physical function (*PF*), role physical (*RP*), bodily pain (*BP*), general health (*GH*), vitality (*VT*), social function (*SF*), role emotional (*RE*) and mental health (*MH*). *p < 0.001; ^#^ *p* < 0.01 using the two-sample Wilcoxon rank-sum (Mann-Whitney) test or Kruskal-Wallis test as appropriate

Significant differences in the scores for individual SF-36 domains were seen in relation to ethnicity, education, damage and active disease manifestations. Patients of Asian ethnicity had higher (better) scores in domains including role physical, bodily pain, general health, vitality, and social function (Fig. 1a; Additional file 1: Table S2). Higher education was also associated with higher domain scores, while the presence of damage, or active musculoskeletal or cutaneous manifestations, were associated with lower (worse) scores across multiple domains (Fig. 1b, c, d; Additional file 1: Table S2). The presence or absence of renal activity did not significantly impact on SF-36 domain scores.

Higher disease activity as measured by the SLEDAI-2 K and PGA, and higher prednisolone dose, were each significantly associated with lower (worse) PCS and MCS scores in simple linear regression analysis (Table 3). With regard to organ domains of disease activity as measured using SLEDAI-2 K, patients with active musculoskeletal manifestations had significantly poorer PCS scores (coeff 0.89, *p* < 0.001), whereas patients with cutaneous manifestations had significantly worse MCS (coeff 0.94, *p* < 0.001). Neither PCS nor MCS scores were significantly different between patients with or without active renal disease. The presence of damage was associated with significantly worse PCS scores, but no differences in MCS scores were observed. Older age at diagnosis (coeff 0.997, *p* < 0.001) and longer disease duration (coeff 0.997, *p* < 0.001) were also associated with poorer PCS but not MCS scores.

**Table 3:** Association of patient and disease characteristics with short form-36 component summary scores in simple linear regression

Variable	Physical component summary			Mental component summary		
	Coeff^*^	*p*	95% CI	Coeff^*^	*p*	95% CI
Country						
Australia	reference	-	-	1.06	0.01	1.01–1.10
China	1.14	<0.001	1.10–1.18	reference		
Indonesia	1.11	<0.001	1.06–1.16	1.06	0.04	1.01–1.12
Philippines	1.23	<0.001	1.18–1.28	1.20	<0.001	1.14–1.26
Singapore	1.14	<0.001	1.10–1.18	1.13	<0.001	1.08–1.18
Taiwan	1.14	<0.001	1.11–1.18	1.05	0.01	1.01–1.10
Thailand	1.12	<0.001	1.09–1.16	1.04	0.10	0.99–1.08
Ethnicity						
Caucasian	reference	-	-	reference	-	-
Asian	1.22	<0.001	1.18–1.26	1.03	0.19	0.99–1.08
Gender						
Female	reference	-	-	reference	-	-
Male	1.02	0.42	0.98–1.06	0.99	0.95	0.95–1.05
Education						
Primary	reference	-	-	reference	-	-
Secondary	1.05	0.001	1.02–1.08	1.01	0.47	0.98–1.05
Tertiary	1.09	<0.001	1.06–1.13	1.06	0.002	1.02–1.09
Age at diagnosis (years)	0.997	<0.001	0.996–0.998	1.00	0.76	0.998–1.001
Disease duration (years)	0.997	<0.001	0.996–0.999	1.00	0.07	0.999–1.003
SLEDAI score	0.994	<0.001	0.992–0.996	0.997	0.03	0.994–0.999
Current organ activity						
CNS	0.82	0.003	0.73–0.93	0.93	0.32	0.79–1.08
Renal	0.98	0.12	0.96–1.00	0.99	0.37	0.96–1.02
MSK	0.89	<0.001	0.86–0.93	0.95	0.03	0.91–0.99
Vasculitis	0.90	0.02	0.83–0.98	0.96	0.46	0.88–1.06
Cutaneous	0.97	0.02	0.94–0.99	0.94	0.001	0.92–0.98
Serositis	0.86	0.01	0.77–0.96	1.04	0.58	0.91–1.18
PGA (0–3)	0.94	<0.001	0.93–0.96	0.94	<0.001	0.93–0.96
Mild flare	0.95	0.002	0.92–0.98	0.98	0.25	0.94–1.02
Severe flare	0.89	<0.001	0.86–0.93	0.95	0.03	0.90–0.99
Prednisolone dose (mg)	0.998	<0.001	0.997–0.999	0.998	<0.001	0.997–0.999
LLDAS	1.04	<0.001	1.02–1.06	1.06	<0.001	1.04–1.09
SLICC-DI score	0.95	<0.001	0.94–0.96	0.99	0.43	0.99–1.01

^*^Coefficient (Coeff) is based on the log-linear model and back-transformed using the exponential function. This represents (coeff-1)*100% increase/decrease in physical component summary or mental component summary scores for change in category (categorical variables), or (coeff-1)*100% change per one unit (continuous variables). Abbreviations: *SLEDAI* systemic lupus erythematosus disease activity index, *MSK* musculoskeletal, *PGA* physician global assessment, *LLDAS* lupus low disease activity state, *SLICC* Systemic Lupus International Collaborating Clinics, *DI* damage index, *CNS* central nervous system

We also analyzed the effect of country of study site and education level as variables. Australian patients recorded the worst PCS scores (43.5, 36.1–52.3), and Chinese patients the worst MCS scores (44.9, 38.5–55.8). In simple linear regression analysis, Asian patients had significantly better PCS scores than their Caucasian counterparts (coeff 1.22, *p* < 0.001) regardless of the country of residence. Both PCS and MCS scores were significantly higher in patients with higher levels of education (Table 3). In backward stepwise multiple linear regression, multiple variables remained significantly associated with PCS (Table 4). The presence of damage remained negatively associated with PCS scores (*p* < 0.001). In contrast, shorter disease duration, younger age at diagnosis, Asian ethnicity, and higher level of education remained significantly positively associated with PCS. Patients with tertiary education (*p* < 0.01) had better MCS scores. The model set-up and properties are shown in Table 4.

**Table 4:** Backward stepwise multiple linear regression for physical component summary (PCS) and mental component summary (MCS)

Model	Variable	Model 1				Model 2				Model 3			
Common variables		PCS		MCS		PCS		MCS		PCS		MCS	
		Coeff	*p*	Coeff	*p*	Coeff	*p*	Coeff	*p*	Coeff	*p*	Coeff	*p*
Model 1	Country												
	Australia	reference	-	1.05	0.10	reference	-	1.04	0.19	reference	-	1.06	0.08
	China	1.04	0.09	reference	-	1.06	*0.02*	reference	-	1.05	*0.04*	reference	-
	Indonesia	1.01	0.73	1.06	*0.04*	1.02	0.40	1.07	*0.03*	1.02	0.45	1.07	*0.02*
	Philippines	1.10	*<0.001*	1.17	*<0.001*	1.12	*<0.001*	1.18	*<0.001*	1.12	*<0.001*	1.19	*<0.001*
	Singapore	1.08	*<0.01*	1.12	*<0.001*	1.09	*<0.001*	1.12	*<0.001*	1.09	*<0.001*	1.13	*<0.001*
	Taiwan	1.06	*0.01*	1.04	0.08	1.07	<0.01	1.03	0.15	1.06	*0.01*	1.03	0.14
	Thailand	1.04	0.11	1.02	0.33	1.05	0.06	1.02	0.39	1.04	0.12	1.02	0.30
	Ethnicity												
	Caucasian	reference	-	reference	-	reference	-	reference	-	reference	-	reference	-
	Asian	1.10	*<0.001*	1.02	0.50	1.10	*<0.001*	1.02	0.62	1.10	*<0.001*	1.02	0.53
	Education												
	Primary	reference	-	reference	-	reference	-	reference	-	reference	-	reference	-
	Secondary	1.03	*0.03*	1.03	0.12	1.03	*0.03*	1.03	0.16	1.03	*0.04*	1.03	0.13
	Tertiary	1.05	*0.002*	1.06	*<0.01*	1.05	*0.001*	1.06	*<0.01*	1.05	*0.001*	1.06	*<0.01*
	Age at diagnosis (years)	0.997	*<0.001*	1.00	0.39	0.997	*<0.001*	1.00	0.39	0.997	*<0.001*	1.00	0.37
	Disease duration (years)	0.998	*0.05*	1.00	0.06	0.998	*<0.01*	1.00	0.08	0.998	*<0.01*	1.00	0.10
	SLICC-DI score	0.96	*<0.001*	0.99	0.13	0.96	*<0.001*	0.99	0.21	0.97	*<0.001*	0.99	0.15
	LLDAS	1.06	*<0.001*	1.05	*<0.001*	-	*-*	-	-	-	-	-	-
Model 2	SLEDAI score	-	-	-	-	0.997	*0.05*	1.00	0.35	-	-	-	-
	PGA (0–3)	-	-	-	-	0.95	*<0.001*	0.97	*0.02*	-	-	-	-
	Mild flare	-	-	-	-	0.98	0.26	0.98	0.25	-	-	-	-
	Severe flare	-	-	-	-	0.97	0.11	0.97	0.33	-	-	-	-
	Prednisolone (mg)	-	-	-	-	0.998	*0.01*	0.999	0.13	-	-	-	-
Model 3	Current organ activity												
	Renal	-	-	-	-	-	-	-	-	1.00	0.43	1.02	0.29
	MSK	-	-	-	-	-	-	-	-	0.93	*<0.001*	0.99	0.55
	Vasculitis	-	-	-	-	-	-	-	-	0.99	0.84	1.02	0.74
	Cutaneous	-	-	-	-	-	-	-	-	0.95	0.70	0.94	*0.04*
	Serositis	-	-	-	-	-	-	-	-	0.96	0.34	1.04	0.49
	PGA (0–3)	-	-	-	-	-	-	-	-	0.99	<*0.001*	0.97	*0.03*
	Mild flare	-	-	-	-	-	-	-	-	0.99	0.59	0.99	0.58
	Severe flare	-	-	-	-	-	-	-	-	0.96	*0.04*	0.98	0.36
	Prednisolone (mg)	-	-	-	-	-	-	-	-	0.998	*<0.01*	0.999	0.19

Variables with *p* values ≤0.1 in simple linear regression analysis were checked for multicollinearity prior to inclusion in the models. To account for different measures of disease activity and disease state that were collinear and to ascertain which of the LLDAS criteria contributed to the relationship with health-related quality of life, three models were used: Model 1 - disease state measured as lupus low disease activity state (LLDAS); Model 2 - breakdown of LLDAS into its individual components/criteria representing measures of disease activity – the systemic lupus erythematosus disease activity index (SLEDAI) score, physician global assessment (PGA), flare index and prednisolone dose; Model 3 - breakdown of SLEDAI score by current organ activity, and PGA, flare index and prednisolone dose. Common independent variables used in all three models are at the top of the table and include: country, ethnicity, education, age at diagnosis, disease duration and Systemic Lupus International Collaborating Clinics (SLICC)-damage index (DI) score. Coefficient (Coeff) is based on log-linear model and back-transformed using the exponential function. This represents (coeff-1)*100% increase/decrease in PCS or MCS scores for change in category (categorical variables), or (coeff-1)*100% change per one unit (continuous variables). Abbreviations: *CNS* (cntral nervous system, *MSK* musculoskeletal. *P* values in italics are significant

### Association between LLDAS or disease activity measures and HR-QoL

**Fig. 2:**
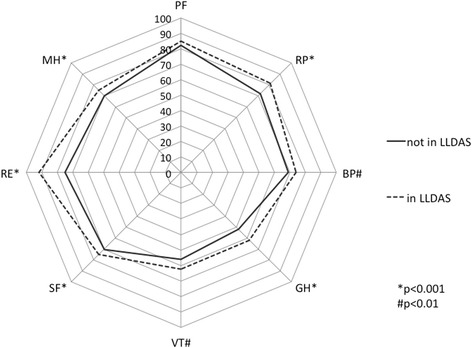
Radar chart comparing short form-36 (SF-36) domain scores between patients in lupus low disease activity state (*LLDAS*) and those not in LLDAS. Each *spoke* on the radar chart represents an SF-36 domain on a scale of 0–100, with higher scores representing better health-related quality of life. The domains are: physical function (*PF*), role physical (*RP*), bodily pain (*BP*), general health (*GH*), vitality (*VT*), social function (*SF*), role emotional (*RE*), and mental health (*MH*). *p < 0.001; ^#^ *p* < 0.01 using the two-sample Wilcoxon rank-sum (Mann-Whitney) test

Patients who fulfilled criteria for LLDAS had significantly higher scores in individual SF-36 domains including role physical, bodily pain, general health, vitality, social function, role emotional and mental health (Fig. 2). The only domain not significantly higher (better) in patients who met the criteria for LLDAS was physical function. Patients in LLDAS also had higher PCS and MCS scores (Table 3). After backward stepwise multiple linear regression adjustment for other variables, patients in LLDAS retained higher PCS scores (*p* < 0.001) and MCS scores (*p* < 0.001) (model 1, Table 4). These findings support the utility of LLDAS and its association with HR-QoL. Analysis of LLDAS individual components in multiple linear regression (model 2, Table 4) showed that a higher SLEDAI-2 K score (*p* = 0.05), PGA (*p* < 0.001) and prednisolone dose (*p* = 0.01) remained negatively associated with PCS scores, whereas disease flares did not have a significant association. Only the PGA (*p* = 0.02) remained significantly negatively associated with MCS scores. Assessing individual organ activity instead of total SLEDAI-2 K score (model 3, Table 4) showed after adjustment that musculoskeletal activity (*p* < 0.001) remained negatively associated with PCS scores, and active cutaneous disease (*p* = 0.04) remained negatively associated with MCS scores.

## Discussion

The ability to define an achievable treatment goal that is predictive of improved outcomes is essential for the implementation of treat-to-target strategies in SLE, and potentially has utility in the analysis of trials of current and novel therapies [19, 31]. Recently, the need to define treatment goals for SLE has received increased attention [20], consequent upon which we reported the definition of a low disease activity treatment outcome state, LLDAS [21]. When disease activity and treatment domains are combined, both of which have been shown to contribute to an adverse long-term outcome in SLE, sustained attainment of LLDAS is associated with protection from accrual of damage over time, as measured using the SLICC-DI, in retrospective analysis of prospectively collected data [21]. Whether LLDAS is associated with measures of HR-QoL has not previously been assessed.

An important finding in the present study is the association between LLDAS and better HR-QoL, even after adjustment for other variables that were associated with HR-QoL. The LLDAS definition represents a composite tool with which patients with clinically diverse phenotypes can be stratified in a binary fashion, as either meeting criteria for LLDAS or not. This “reductionistic” approach takes advantage of the fact that the heterogeneity of disease expression in active SLE is, by definition, lessened as the disease activity lessens [18]. By combining different measures of clinical activity, and those of medication burden, the LLDAS is an encompassing measure of the overall clinical state of the patient, and emerging data confirm that the domains of LLDAS contribute independently to the stringency of the measure [32]. This means that LLDAS, rather than simply representing a description of mild disease, represents a composite treatment target state. Non-attainment of LLDAS could therefore reflect flare, refractory disease or insufficient treatment intensity, just as is the case with low disease activity definitions in RA. Given that improvement in HR-QoL is recognized as an important outcome measure in clinical trials [3, 8], the association between LLDAS and better SF-36 scores further supports its utility as a treatment target. Prospective studies showing that attainment of LLDAS is associated with improvements in HR-QoL over time are required, and are in progress.

In order to scrutinize the effects of the LLDAS components on HR-QoL, we utilized separate multiple linear regression models. SLEDAI-2 K, PGA and prednisolone dose (potentially a surrogate for activity) were each significantly and negatively associated with PCS scores, but only the PGA was negatively associated with MCS scores. Interestingly, disease flares as measured by the SFI were not significantly associated with either PCS or MCS scores. Of note, due to the cross-sectional nature of the analyses in this study, the SFI was used as a surrogate for the third criterion of LLDAS, which is that there must be no new features of lupus disease activity compared to the previous assessment [21]. It is possible that with longitudinal analysis, this LLDAS criterion may be significantly associated with HR-QoL.

The relationship between disease activity and HR-QoL in SLE remains controversial in the published literature [12, 25, 33–35], likely due to a combination of varying study designs, an inherently heterogeneous disease, different measures of activity and fluctuating disease states. Our study is the first to analyze HR-QoL in relation to individual organ system activity based on the SLEDAI. We observed a negative association between active musculoskeletal disease and poorer PCS, and between active cutaneous disease and poorer MCS scores. We consider that it makes clinical sense that active joint and muscle disease affects physical function, while cutaneous disease influences mental wellbeing; young women with SLE who comprise the majority of patients are known to suffer from poor body image [36]. An effect of renal activity on HR-QoL has been described by Appenzeller et al., who reported that patients with active renal disease had slightly poorer physical function, albeit with wide confidence intervals [37]. In contrast we found no significant association between active renal disease and any domains of the SF-36. Some organ involvement, such as lupus nephritis, may be inherently clinically silent in terms of HR-QoL, despite reflecting a serious threat to health.

Although undertaken in order to evaluate the association between LLDAS and HR-QoL, this is one of the largest studies to date of HR-QoL in patients with SLE, and as such it affords the opportunity to investigate other factors associated with HR-QoL in SLE. Patient characteristics, such as ethnicity, have previously been shown to be associated with various aspects of disease burden in SLE [38, 39], with Caucasian patients having lower disease activity but reporting poorer HR-QoL compared to their non-Caucasian counterparts [35, 40]. Studies from individual countries within the Asia Pacific region report poorer HR-QoL in patients with SLE compared to national averages [33], and negative associations with poorer socioeconomic status [26]. However, to date, between-country comparisons have been lacking.

We have demonstrated important regional and ethnic differences in HR-QoL. In our study, compared to Caucasians, patients of Asian ethnicity reported better PCS, even when adjusted for other variables, but no significant differences in MCS scores. Similar findings have been reported in different ethnic groups in Canada and the USA, with white ethnicity associated with poorer physical, but not mental function [4, 35]. The SF-36 has been cross-culturally validated to allow global comparisons, but it is unlikely that it is sensitive to all cultural and ethnic nuances. The significant difference in PCS and MCS scores between countries in our cohort, even when adjusted for ethnicity and disease factors, further highlights the importance of cultural differences in perception of the impact of disease and patients’ coping strategies, which have been suggested to be just as important as disease states in determining HR-QoL in SLE [41]. The ability to cope better with illness was potentially reflected in the association between higher education and better summary scores, a finding supported by previous studies [4, 33]. However, this may also be indicative of patients with higher levels of education being employed in less manually labor-intensive jobs, therefore with potentially a less noticeable impact on physical function.

Studies assessing the association between organ damage and HR-QoL have reported discrepant results. We identified significant association between greater damage and PCS scores, but not MCS scores, which is also seen in the ethnically diverse LUMINA cohort [4]. Similarly, in a longitudinal study of Chinese patients from Hong Kong, Mok et al., showed that accrual of new damage predicted a decline in SF-36 scores [33]. In contrast, in a predominantly Caucasian population with low damage accrual over 8 years, no disease features were associated with decline in physical functioning except for the presence of fibromyalgia [35].

The lack of measurements to identify fibromyalgia and other comorbidities is one of the limitations of this study, as pain and fatigue have been shown to independently influence HR-QoL in patients with SLE [6, 10, 11]. Two domains of the SF-36, bodily pain and vitality, are potential surrogate measures for pain and fatigue respectively. Patients in LLDAS had significantly higher (better) scores in both of these domains, with the inference that LLDAS may be associated with a reduction in pain and fatigue. A disease-specific HR-QoL tool could further address the additional issues pertinent to patients with SLE and assess the effect of LLDAS on these; however, the currently available disease-specific instruments have not been validated in all the spoken languages of this multicultural cohort of patients.

Additionally, clear evidence of superiority is lacking among the multiple disease-specific HR-QoL tools [5]. The cross-sectional nature of the analyses does not allow the assessment of changes in HR-QoL with fluctuating disease states. However, given that the SF-36 is designed to capture HR-QoL in the preceding 4 weeks, the same time frame as the evaluation of disease activity, it should be relevant to disease activity measures captured at the same time. A longitudinal study is underway, which will enable analysis of the association between LLDAS and transitions in HR-QoL measured by the SF36. Assessment of the effect of LLDAS on other PRO measures, such as patient assessment of disease activity, could form the basis of future validation studies.

## Conclusions

In summary, we have shown for the first time that LLDAS is associated with better HR-QoL. This supports the validity of this definition of treatment outcome state for potential use in clinical practice, treat-to-target studies and clinical trials. This conclusion would be further supported by longitudinal studies, of which at least one is underway. In addition, we have described important ethnic, socioeconomic and disease-specific associations with HR-QoL in one of the largest multiethnic SLE cohorts ever studied. Attention to reversible or preventable precipitants of poor HR-QoL should be included in the management of SLE.

## Additional file

Additional file 1: Table S1. Disease manifestations ever present. **Table S2.** Comparison of SF-36 domain scores by patient and disease characteristics (DOCX 19 kb)

## Abbreviations

ACR: American College of Rheumatology
APLC: Asia Pacific Lupus Collaboration
BP: Bodily pain
CNS: Central nervous system
coeff: Coefficient
DI: Damage index
GH: General health
HR-QoL: Health-related quality of life
LLDAS: Lupus low disease activity state
MCS: Mental component score
MH: Mental health
MSK: Musculoskeletal
PCS: Physical component score
PF: Physical function
PGA: Physician global assessment
PRO: Patient-reported outcome
RE: Role emotional
RP: Role physical
SELENA: Safety of Estrogens in Lupus Erythematosus National Assessment
SF: Social function
SFI: Safety of Estrogens in Lupus Erythematosus National Assessment- systemic lupus erythematosus Erythematosus flare index
SLE: Systemic lupus erythematosus
SLEDAI: Systemic lupus erythematosus disease activity index
SLICC: Systemic Lupus International Collaborating Clinics
VT: Vitality
