# Supplementary material for: Association of the lupus low disease activity state (LLDAS) with health-related quality of life in a multinational prospective study
Source: Arthritis Res Ther. 2017 Mar 20;19:62. doi: 10.1186/s13075-017-1256-6 (PMC5359963; doi:10.1186/s13075-017-1256-6)
Supplement: Additional file 1: Table S1. — Disease manifestations ever present. Table S2. Comparison of SF-36 domain scores by patient and disease characteristics (DOCX 19 kb) [file 13075_2017_1256_MOESM1_ESM.docx]

**Table S1: Disease manifestations ever present**

|  | n (%) or mean (SD) |
| --- | --- |
| ACR criteria  Malar rash  Discoid rash  Photosensitivity  Mouth Ulcers  Arthritis  Serositis  Renal  Neurologic  Haematologic  Immunologic  ANA | 832 (58%)  230 (16%)  423 (30%)  532 (37%)  892 (63%)  248 (17%)  654 (46%)  125 (9%)  877 (62%)  1164 (82%)  1301 (91%) |
| SLICC Criteria  Acute cutaneous lupus  Chronic cutaneous lupus  Oral ulcers  Non-scarring alopecia  Synovitis  Serositis  Renal  Neurologic  Hemolytic anemia  Leukopenia  Thrombocytopenia  ANA  Anti-dsDNA  Anti-Sm  Antiphospholipid antibodies  Hypocomplementaemia  Positive Coomb’s in absence of heamolysis | 959 (67%)  259 (18%)  538 (38%)  364 (26%)  883 (62%)  249 (17%)  655 (46%)  151 (11%)  276 (19%)  735 (52%)  196 (14%)  1301 (91%)  1052 (74%)  289 (20%)  310 (22%)  845 (59%)  74 (5%) |
| Number of ACR criteria | 5 .10 (1.38) |
| Number of SLICC criteria | 6.28 (1.84) |

ACR Criteria: Arthritis (two or more joints with tenderness, swelling or effusion), serositis (pleuritis or pericarditis), renal disorder (persistent proteinuria >0.5grams per day, or presence of cellular casts), neurologic disorder (seizures or psychosis not attributable to other causes), haematologic disorder (haemolytic anaemia, leukopaenia, lymphopaenia or thrombocytopaenia), immunologic criteria (presence of anti-dsDNA antibody, anti-Sm antibody, or positive finding of antiphospholipid antibodies).

SLICC Criteria: Acute cutaneous lupus (lupus malar rash, bullous lupus, toxic epidermal necrolysis, maculopapular lupus rash, photosensitive lupus rash, subacute cutaneous lupus), chronic cutaneous lupus (classic discoid rash, hypertrophic lupus, lupus panniculitis, mucosal lupus, lupus erythematosus tumidus, chilblains lupus), synovitis (involving 2 or more joints), serositis (pleuritis or pericarditis), renal (urine protein creatinine ratio 500mg/24hrs or more, or presence of red cell casts), neurologic (seizures, psychosis, mononeuritis multiplex, myelitis, peripheral or cranial neuropathy, acute confusional state).

Abbreviations: SLE (systemic lupus erythematosus); ACR (American College of Rheumatology); SLEDAI (SLE disease activity index); SLICC (Systemic Lupus International Collaborating Clinics); DI (damage index); ANA (antinuclear antibody); ds-DNA (double stranded DNA)

**Table S2: Comparison of SF-36 domain scores by patient and disease characteristics**

|  | Ethnicity | | | Education | | | | Disease damage | | | MSK activity | | | LLDAS | | |
| --- | --- | --- | --- | --- | --- | --- | --- | --- | --- | --- | --- | --- | --- | --- | --- | --- |
|  | Caucasian | Asian |  | Primary | Secondary | Tertiary |  | No damage | Some damage |  | MSK inactive | MSK active |  | Not in LLDAS | In LLDAS |  |
|  | Median (IQR) | Median (IQR) | *p** | Median (IQR) | Median (IQR) | Median (IQR) | *p^#^* | Median (IQR) | Median (IQR) | *p** | Median (IQR) | Median (IQR) | *p** | Median (IQR) | Median (IQR) | *p** |
| PF | 65 (42.5-90) | 85 (70-95) | <0.001 | 80 (55-90) | 85 (69-95) | 90 (75-95) | <0.001 | 90 (75-95) | 75 (50-90) | <0.001 | 85 (70-95) | 80 (55-90) | <0.001 | 82 (65-95) | 85 (97.5-95) | 0.12 |
| RP | 50 (25-93.8) | 75 (50-100) | <0.001 | 68.8 (34.4-93.8) | 75 (50-96.9) | 81.3 (56.3-100) | <0.001 | 75 (56.3-100) | 68.8 (37.5-93.8) | <0.001 | 75 (50-100) | 56.3 (37.5-81.3) | <0.001 | 71.9 (43.8-93.8) | 81.3 (62.5-100) | <0.001 |
| BP | 51 (31-75) | 74 (52-84) | <0.001 | 62 (41-84) | 67 (51-84) | 74 (62-84) | <0.001 | 74 (52-84) | 64 (41-84) | <0.001 | 74 (52-84) | 52 (31-74) | <0.001 | 69 (51-84) | 74 (52-84) | <0.01 |
| GH | 41 (25-62) | 57 (40-72) | <0.001 | 52 (35-67) | 55 (37-70) | 57 (42-72) | <0.01 | 57 (40-72) | 52 (35-67) | <0.001 | 57 (40-72) | 47 (30-67) | <0.001 | 52 (35-67) | 62 (42-75) | <0.001 |
| VT | 43.8 (25-60) | 62.5 (50-76) | <0.001 | 55 (43.8-68.8) | 56.3 (43.8-70) | 65 (50-75) | <0.001 | 62.5 (50-75) | 56.3 (43.8-68.8) | <0.001 | 62.5 (50-75) | 50 (37.5-75) | <0.001 | 56.3 (43.8-75) | 62.5 (50-75) | <0.01 |
| SF | 62.5 (37.5-87.5) | 75 (50-100) | <0.01 | 75 (50-100) | 75 (50-100) | 75 (50-87.5) | 0.36 | 75 (50-100) | 75 (50-100) | 0.09 | 75 (50-100) | 62.5 (50-75) | <0.001 | 70 (50-87) | 75 (50-100) | <0.001 |
| RE | 83.3 (50-100) | 83.3 (58.3-100) | 0.71 | 75 (33.3-100) | 75 (50-100) | 83.3 (66.7-100) | <0.001 | 83.3 (58.3-100) | 75 (50-100) | <0.01 | 83.3 (58.3-100) | 66.7 (33.3-91.7) | <0.001 | 75 (50-100) | 91.7 (66-7-100) | <0.001 |
| MH | 70 (50-85) | 70 (56-80) | 0.95 | 68 (55-80) | 70 (55-80) | 75 (60-80) | <0.001 | 70 (56-80) | 70 (56-80) | 0.45 | 70 (56-80) | 70 (55-80) | 0.25 | 70 (55-80) | 75 (60-80) | <0.001 |

**p*-value calculated using Wilcoxon rank-sum (Mann-Whitney) test

^#^*p*-value calculated using Kruskal-Wallis test

Abbreviations: MSK (musculoskeletal); LLDAS (Lupus Low Disease Activity State); PF (physical function); RP (role physical); BP (bodily pain); GH (general health); VT (vitality); SF (social function); RE (role emotional); MH (mental health).
